# Ultrasonic Characterization of Components Manufactured by Direct Laser Metal Deposition

**DOI:** 10.3390/ma13112658

**Published:** 2020-06-11

**Authors:** Anna Castellano, Marco Mazzarisi, Sabina Luisa Campanelli, Andrea Angelastro, Aguinaldo Fraddosio, Mario Daniele Piccioni

**Affiliations:** 1 Department of Civil Engineering Sciences and Architecture, Polytechnic University of Bari, Via Orabona 4, 70125 Bari, Italy; aguinaldo.fraddosio@poliba.it (A.F.); mariodaniele.piccioni@poliba.it (M.D.P.); 2 Department of Mechanics, Mathematics and Management, Polytechnic University of Bari, Via Orabona 4, 70125 Bari, Italy; sabinaluisa.campanelli@poliba.it (S.L.C.); andrea.angelastro@poliba.it (A.A.)

**Keywords:** additive manufacturing, direct laser metal deposition, ultrasonic immersion tests

## Abstract

Direct laser metal deposition (DLMD) is an innovative additive technology becoming of key importance in the field of repairing applications for industrial and aeronautical components. The performance of the repaired components is highly related to the intrinsic presence of defects, such as cracks, porosity, excess of dilution or debonding between clad and substrate. Usually, the quality of depositions is evaluated through destructive tests and microstructural analysis. Clearly, such methodologies are inapplicable in-process or on repaired components. The proposed work aims to evaluate the capability of ultrasonic techniques to perform the mechanical characterization of additive manufactured (AM) components. The tested specimens were manufactured by DLMD using a nickel-based superalloy deposited on an AISI 304 substrate. Ultrasonic goniometric immersion tests were performed in order to mechanical characterize the substrate and the new material obtained by AM process, consisting of the substrate and the deposition. Furthermore, the relationship was evaluated between the acoustic and the mechanical properties of the AM components and the deposition process parameters and the geometrical characteristics of multiclad depositions, respectively. Finally, the effectiveness of the proposed non-destructive experimental approach for the characterization of the created deposition anomalies has been investigated.

## 1. Introduction

As defined by the American Society for Testing and Materials International (ASTM), additive manufacturing (AM) is a manufacturing process in which the components are produced by addition and not subtraction of material [1]. The degree of maturity of certain technologies is opening an interesting horizon in the new fields of mold production, jewelry and art, beyond leading sectors such as the aerospace and biomedical industries. These kinds of technologies rely on 3D CAD models that recreate the whole geometry of the part. The manufacturing processes are carried out with a “near-net shape” logic of several layers [2,3,4].

The AM technologies are classified basing on the feedstock material (metal, polymer and ceramic) and the equipment used [5]. Metal AM processes are the most challenging due to the intrinsic characteristics of the materials that require high intensity heat sources and are categorized according to the material feeding methods [6]. The technologies that adopt wire feed systems and an arc or laser as heat source have high deposition rates and low dispersion of environmental pollutants [7,8]. On the other hand, feeding the material in the form of powder allows for much more flexible processing. The powder bed technologies produce components with innovative structural designs, which are impossible to achieve using traditional methods. These systems can also process reactive materials under vacuum conditions by means of a laser or an electron beam [9,10,11]. The direct powder feed systems, such as direct laser metal deposition (DLMD), are used in several industrial applications for cladding, 3D manufacturing or repairing worn components. The selection of parameters, deposition strategies and process conditions strongly influence the geometric accuracy, the surface finish and the mechanical properties of the final component [12,13]. An erroneous design of the processes can lead to the appearance of the typical AM deposition flaws such as porosity, voids, cracks and lack of fusion [14]. In sectors that provide very high-performance standards, the presence of these defects can lead to non-acceptance of the final component. Currently, the related normative framework is in development, in order to enable the certification of products created by AM technologies. 

The assessment of the quality of the AM components is a crucial issue and different experimental solutions have been proposed in the literature to solve this problem. 

Non-destructive techniques are currently the experimental procedures that allow the inspection and the identification of the flaws during and after the deposition process. Among these techniques, the capabilities of ultrasonic testing have recently been investigated by researchers to develop reliable procedures for the process monitoring and the quality control of AM components [15,16]. In particular, in Sol, T. et al. [15] the pulse-echo ultrasonic method was performed in order to investigate the mechanical behavior of selective laser melting additively manufactured AlSi_10_Mg. A slight elastic anisotropy with symmetry along the build direction was identified by means of ultrasonic tests. The anisotropy has been related to the attenuation of the experimental data in terms of velocity and frequency of the ultrasonic waves. 

The potentiality of Non Destructive Testing (NDT) techniques for in-process and off-line inspections of wire and arc additive manufacturing (WAAM) parts was investigated in [16], proving the limitations of the ultrasonic tests regarding the necessity of a proper surface finishing of the specimens. This drawback can be overcome by using ultrasonic immersion techniques [17], which involve non-contact transducers, as showed in the proposed work. 

A crucial problem that compromises the interpretation of ultrasonic data is the structural noise in the ultrasonic waveforms, which interferes with the wave reflections caused by defects. The structural noise is known as ultrasonic backscattering signal and contains meaningful information on the geometric and mechanical properties of the material microstructure. In [18] the ultrasonic backscattering data generated by the microstructure of the AM material produced by selective laser melting (SLM) process were evaluated using the ultrasonic immersion C-scan system.

Another non-contact ultrasonic method was proposed in Cerniglia et al. [19], consisting of the innovative laser ultrasonic technique that was employed for the inspection of laser powder deposition (LPD) components in order to detect near-surface and surface defects. The authors proposed a prototype laser system mounted on the LPD robot, which allows for accurate inspections of the LPD parts during the AM manufacturing.

Nonlinear ultrasonic techniques [20,21] were applied in [22] to study the dislocation density produced in AM components made through powder bed fusion (PBF) and laser engineered net shaping (LENS) techniques. Here, an interesting experimental setup and a promising theoretical model for the quantification of microstructural changes based on the analysis of the higher order harmonics of surface waves that propagate in the specimens were proposed [23]. The Rayleigh waves were generated on the surface of an AM component by a traditional contact transducer coupled to a wedge and were received by an air-coupled transducer (a non-contact ultrasonic probe). 

While on the one hand the ultrasonic method is promising both for the identification of defects and for the mechanical characterization of the AM components, it is still a great challenge for researchers, for the resolution of some issues related to the interpretation of the ultrasonic signals and the development of suitable theoretical models that correctly describe the mechanical behavior of the materials deriving from the AM process. However, there are still few studies in literature that propose theoretical and experimental procedures to solve the issue of mechanical characterization of the AM components. 

Here, an experimental approach to the mechanical characterization of AM components based on the results obtained by ultrasonic goniometric immersion tests is proposed. The aim is to employ the proposed experimental approach for the mechanical characterization of the whole component, both of the substrate and of the new material obtained by the AM process, consisting of the substrate and the deposition. 

Furthermore, the suggested experimental method can also allow the characterization of multilayer depositions which have an anisotropic mechanical response [24], as well as the evaluation of residual stress states [25] generated during the production of the AM components.

Ultrasonic goniometric immersion tests were performed on specimens made by direct laser metal deposition (DLMD), depositing a nickel-based superalloy on an AISI 304 substrate. The specimens differ in the percentage of the clad overlap (OV), as well as for the height of the depositions and the depth penetrations in the substrate. Moreover, a slight perturbation of the powder flow was induced in the deposition of two specimens in order to evaluate the effectiveness of the proposed experimental approach for the characterization of the deposition anomalies.

Starting from the measurement of the times of flight (TOF) of the ultrasonic waves in the substrate and in new materials (substrate + deposition) obtained by the AM process, the ultrasonic velocities of the longitudinal and transversal waves were evaluated, respectively. By exploiting the measurements of the ultrasonic velocities, using the theoretical model of the isotropic material, the elastic moduli that characterize the mechanical behavior of the substrate and of the new resulting material were determined. 

By means of the proposed experimental approach, the variation of the acoustic response (in terms of TOF and of the velocity of the ultrasonic waves) and of the mechanical response (in terms of the elastic moduli) of the AM components was evaluated as a function of the deposition process parameters and of the geometrical characteristics of multiclad depositions (particularly the average clad height). 

Finally, the capability of the proposed ultrasonic approach to characterize the deposition anomalies were investigated by discussing the ultrasonic test results in terms of the TOF and velocity of ultrasonic waves and also of the elastic moduli. 

The outcomes shown are the first results obtained by using the proposed non-destructively experimental approach. Further experimental campaigns are ongoing in order to mechanical characterize the AM components that exhibit anisotropic mechanical behavior.

## 2. Materials and DLMD Machine Setup 

In this work, several specimens were created using DLMD. The heat source is a fiber laser with a nominal power of 4 kW and wavelength of 1.070 µm (YLS 4000 IPG Photonics Ytterbium Laser System Cambridge, MA, USA), focused on a substrate in order to create a molten pool.

The powder coming from an external powder feeder is carried by means of Argon gas and conveyed inside the pool to build a solid trace. The gas was also employed as shielding element to prevent clad oxidation. A 5-axis machine equipped with a deposition head and a coaxial nozzle was used. By moving the head along a specific path, it is possible to build complex geometries.

The AISI 304 (Table 1) substrate is an austenitic stainless steel with a good resistance to corrosion in the presence of chlorides. The nickel as alloying element allows a good dilution and an acceptable mixing between the added material and the substrate, the latter with dimensions of 100 × 80 mm and a thickness of about 6 mm.

The nickel-based superalloys (Table 2) sport exceptional performances at high temperature (1000 °C) for extended periods, which makes them suitable for high-performance applications, but are remarkably difficult to manufacture. The granulometry of the powder used is in the range 15–45 µm.

In the process design, parameters such as laser power (P), spot diameter (D), travel speed (V) and powder feed rate (F) were defined. The specimens were built applying different sets of process parameters identified in previous studies as feasible for a deposition with good characteristics as shown in Table 3.

A unidirectional “raster” strategy was selected for the multiclad deposition, in which 5 single tracks with a length of 40 mm were placed side by side. Fundamental feature in multiclad deposition is clad overlap (OV%) that define the percentage of intersection of adjacent tracks [26].

The experiment has been carried out with an overlap equal to 10% (S1), 20% (S2), 30% (S3), 40% (S4) and 50% (S5). Moreover, in order to evaluate the capability of the proposed experimental approach to characterize some deposition anomalies in the AM components, a slight perturbation of the powder flow was induced in the deposition S2 and S4. In this way, different substrate penetration depths and interlayer porosity issues were obtained. Two replications were carried out for each set of parameters.

**Figure 1:**
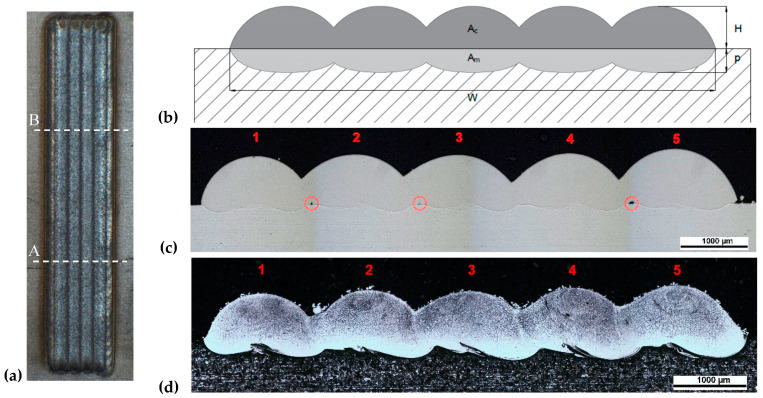
Front view of deposition 3.2 with cross-section positions (**a**); schematic representation of a deposition with geometrical characteristics (**b**); macrographic cross-section of deposition 1.3 (**c**); macrographic cross-section after chemical etching of deposition 2.3 (**d**).

Subsequently, the specimens were trimmed using a SiC cut-off wheel in position A and B (see Figure 1a), hot mounted in phenolic resin, polished and chemically etched. To examine the cross-section geometry and ensure the presence of flaws, a Nikon Eclipse MA200 inverted optical microscope (Tokyo, Japan) optimized for digital imaging, was used.

The geometrical characteristics considered in the analysis are shown in the schematic representation of the deposition in Figure 1b. The scheme is compared with a macrographic cross-section images of the depositions (Figure 1c) in which the presence of the flaws at the interface between deposition and substrate are indicated. The macrographic cross-section images of the depositions after etching (Figure 1d) are useful to emphasize the penetration on the substrate of the process. This parameter is of paramount importance because, as it grows, the bond between the base material and the cladding increases. However, the mixing between the two materials also increases, changing the metallurgical and mechanical characteristics of the same. A too low penetration could cause the cladding detachment, while a too high value would compromise the characteristics of the component.

Table 4 lists the tested AM specimens with the relative denomination, the percentage of overlap (OV%), and the geometrical characteristics of width (W), clad area (A_c_) and substrate molten area (A_m_) of each deposition. Moreover, the average clad height (H_avg_) and the average substrate penetration (p_avg_) were calculated as a ratio between relative areas and the width. Finally, the dilution (Dil) computed as ratio between the average substrate penetration and the height of the deposition (H_avg_ + p_avg_). The values reported in Table 4 are the average of the measurements taken for each cross-sections of replications.

**Figure 2:**
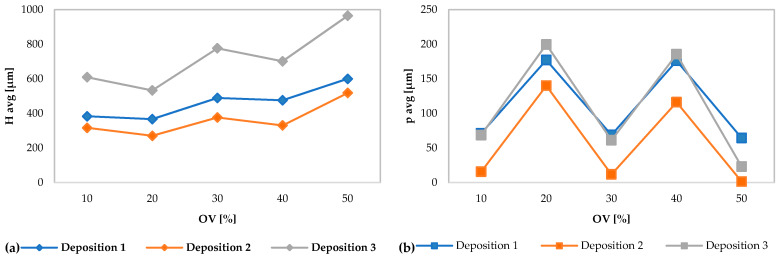
Geometric characteristics of average clad height (**a**) and average substrate penetration (**b**) for each deposition vs overlap percentage.

In Figure 2a, the H_avg_ and p_avg_ are plotted with respect the overlap percentage of each specimen. The progressively closer depositions cause an increase in the average height of the clad. In Figure 2b it is possible to observe the strong variations of p_avg_ in the depositions with an overlap ratio of 20% and 40% which are derived from the imposed perturbation of the powders flow.

## 3. Ultrasonic Goniometric Immersion Tests

### 3.1. Theoretical Model 

From a theoretical point of view, the wave phenomena involved in the ultrasonic test can be viewed as small perturbation of an initial state of a body. Thus, it is possible to study the propagation of ultrasonic waves by applying the linear theory of elastodynamics [24,27]. 

In particular, the propagation of plane progressive elastic waves may be described by assuming a displacement field:

(1) u(x,t)=a φ(x⋅n-v t),

where **x** is a point of referential configuration of the body at the time t, **a** is the direction of motion, **n** is the direction of wave propagation, v is the velocity of propagation and φ is a real valued smooth function.

In absence of the body forces, the wave propagation is ruled by the equation of the motion:

(2) $$\mathrm{Div}\left(C\left[\nabla u\right]\right)=\rho \ddot{u},$$

where ρ=ρ(x) is the mass density and C=C(x) is the incremental fourth order elasticity tensor referred to the initial state of the body. 

From Equation (2), it is possible to write the condition for wave propagation (the classical Fresnel–Hadamard’s propagation condition) in the form of the Christoffel equation:

(3) $$\left[\Gamma \left(n\right)\;-\;\rho v^{2}I\right]\;a=o,$$

where Γ(n) is the second order Kelvin–Christoffel propagation tensor for the direction **n**, given by:

(4) $$\Gamma \left(n\right)=C^{t}\left[n\;⊗\;n\right],$$

where the superscript “t” represent the minor transposition for fourth order tensors. 

Equation (4) shows that the Christoffel tensor Γ(n) is related only to the elasticity tensor C and to the direction of propagation **n**. 

Moreover, if an elastic wave propagates in a certain direction **n**, the Christoffel Equation (3) shows that the square of the wave velocity v is an eigenvalue of the Christoffel tensor for the direction of propagation **n**, while the direction of motion **a** is the related eigenvector. Thus, the symmetry properties of the material strongly influence the features of progressive elastic waves: so, by Equations (3) and (4), the elastic constants (i.e., the components of C) are linked to the velocity of propagation along a fixed direction **n**.

The Federov–Stippes theorem demonstrates that if the elasticity tensor C is symmetric and strongly elliptic, then at a point **x** there exist longitudinal (**a** and **n** parallel) and transverse (**a** and **n** perpendicular) elastic waves [24]. If the material is isotropic, for each possible direction of propagation **n** only “pure” propagation modes like longitudinal waves and transverse waves may propagate. On the contrary, for anisotropic materials in a generic direction of propagation **n**, different from an axis of material symmetry, elastic waves may propagate in a “not pure” propagation mode (as quasi-longitudinal waves or as quasi-transverse waves). 

This theoretical framework suggests a non-destructive ultrasonic experimental characterization of the materials. Then, the results of ultrasonic tests can be employed for solving two major problems in the mechanics of elastic materials: (1) “the classification problem”, that is the determination of the symmetry class and the identification of the material symmetry axes; (2) the “representation problem”, that is, once known the symmetry class, the determination of the components of the elastic tensor characterizing the elastic response of the material. 

In the study case, adopting the theoretical model of the isotropic material for describing the mechanical response of the AM components, it was possible to determine the elastic moduli starting from experimental measures of the ultrasonic wave velocities. 

To this aim, the inversion of the Christoffel Equation (3) has yielded the well-known relations in Equation (5):

(5) $$E=\rho V_{T}^{2}\frac{{3V}_{L}^{2}-{4V}_{T}^{2}}{V_{L}^{2}-V_{T}^{2}},\;G=\rho V_{T}^{2},\;\nu =\frac{{\left(\frac{V_{L}^{}}{V_{T}^{}}\right)}^{2}-2}{2\left[{\left(\frac{V_{L}^{}}{V_{T}^{}}\right)}^{2}-1\right]},$$

between the engineering elastic moduli (the Young’s modulus E, the shear modulus G and the Poisson’s ratio ν), the velocity of longitudinal waves V_L_ and of longitudinal waves V_T_ and the density ρ. 

### 3.2. Ultrasonic Tests Setup

The ultrasonic tests were performed by Laboratorio Ufficiale Prove Materiali “M. Salvati” of Polytechnic University of Bari, a laboratory equipped with advanced facilities for non-destructive testing [24,25].

**Figure 3:**
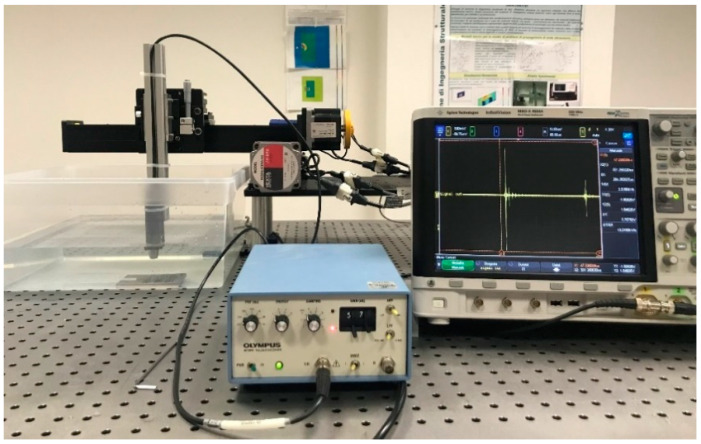
Ultrasonic immersion tests setup: main components.

The experimental setup (Figure 3) consists of:

- An ultrasonic immersion focused transducer (central frequency equal to 5 MHz, Olympus NDT, Waltham, MA, USA) connected to a platform which allow a 3-DOF motion and a rotation of the probe according to fixed angles;
- An Olympus ultrasonic pulser/receiver 5072PR (Olympus NDT, Waltham, MA, USA) for generating and receiving the ultrasonic waves;
- A digital oscilloscope Agilent MSU-X-4054A (500 MHz, 4 channels, Agilent, Santa Clara, CA, USA) for monitoring the ultrasonic signals;
- A water tank;
- A workstation with ad hoc developed software in Labview (2017) for each phase of tests: the management of the ultrasonic probe and the reprocessing of the experimental data.

Unlike the contact tests, in the ultrasonic immersion tests the sample and the transducers are immersed in a water tank without any contact. In this case, the water acts as a coupler allowing an optimal acoustic coupling. 

Furthermore, the movement of the probe is not manual, as it is in contact tests, but automatic.

**Figure 4:**
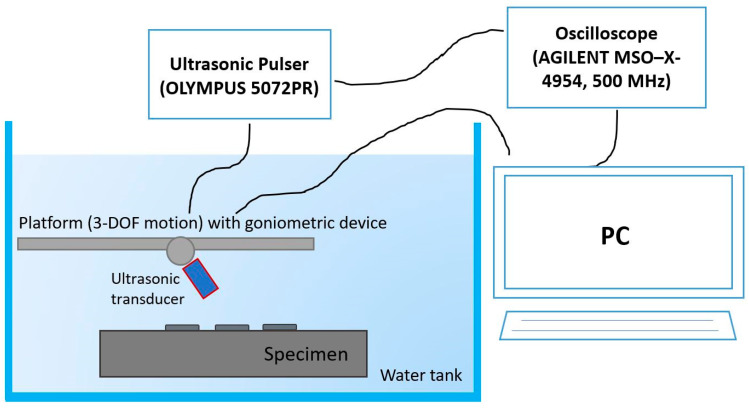
Ultrasonic immersion tests setup: a schematic drawing showing the rotation of the ultrasonic transducer by means of the goniometric device.

The two characteristics above make the experimental measurements of the ultrasonic velocities more accurate. Not only, it is possible thanks to the rotation of the ultrasonic probe (or the sample) to investigate the acoustic response of the material according to different angles of inclination of the ultrasonic beam respect to the surface of the sample (Figure 4). This makes the goniometric immersion ultrasonic tests the best non-destructive experimental solution for the mechanical characterization of anisotropic materials, as the acoustic (mechanical) response varies according to the direction of propagation of the ultrasonic waves.

Notice that the water supports only the longitudinal waves, but the transverse waves can be generated in the sample by the change of the polarization of the acoustic wave crossing the interface between different media, i.e., by the mode conversion in according to the Snell law

(6) $$\frac{\sin\theta_{i}}{v_{w}}=\frac{\sin\theta_{r}}{v_{p}},$$

where $\theta_{i}$ is the angle of incidence on the surface of the sample of the ultrasonic beam travelling in the water, $v_{w}$ is the ultrasonic velocity in the water, $v_{p}$ is the phase velocity of the refracted beam into the sample and $\theta_{r}$ is the angle of refraction of the ultrasonic beam into the sample.

**Figure 5:**
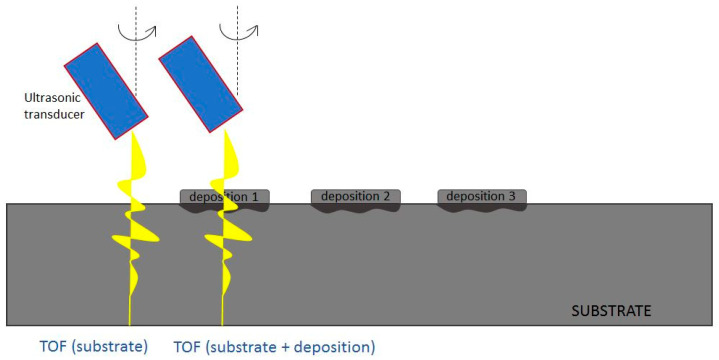
Experimental procedure for measuring times of flight (TOF) of the ultrasonic waves in substrate and in the resulting additive manufactured (AM) material (substrate + deposition) for each specimen.

The tests were performed by using the pulse-eco technique (or back reflection technique), i.e., a single probe is employed that emits and receives the ultrasonic signal. During the tests, the TOF of the ultrasonic waves that propagate in the sample by rotating the probe according to given angles was determined. In particular, the TOF of ultrasonic waves propagating into the substrate and the TOF of the ultrasonic waves propagating into the new material resulting from the AM process, consisting of the substrate and of the deposition, for each specimen, respectively, were measured (Figure 5). 

It is pointed out that the TOF measurements were carried out through an averaging process of the acquired and normalized ultrasonic signals for each area of the tested AM specimens, performed by the ad hoc developed software in Labview.

Therefore it must be stressed that the measurements of the TOF of the longitudinal waves both in the substrate and in the substrate + deposition of each specimen were carried out with a probe rotation angle equal to 0° (i.e., the probe position is orthogonal to the sample surface), which corresponds to the maximum energy of the received ultrasonic pulse. Instead, the measurement of the TOF of the transverse waves was carried out with an angle greater than the first critical angle. In particular, the Labview software enabled the identification of the angle at which the wave energy of transversal waves is maximum (i.e., the angle corresponding to the maximum amplitude of the measured shear waves). In the case of the transversal waves, the probe rotating angles range was 15.00°–16.00° both in the substrate and in the deposition + substrate of each specimen.

Starting from the TOF of the ultrasonic waves, knowing the thickness of the substrate and of the new material (substrate + deposition), the ultrasonic velocities of the longitudinal waves and of the transversal waves were determined. Finally, knowing the density of the substrate and the resulting AM material (substrate + deposition), the elastic moduli were determined through the equations in (5). 

### 3.3. Ultrasonic Test Results: the Time of Flight and the Velocity of the Ultrasonic Waves 

The tests performed on each specimen have enabled us to determine the TOF of the ultrasonic longitudinal and transversal waves, respectively.

**Figure 6:**
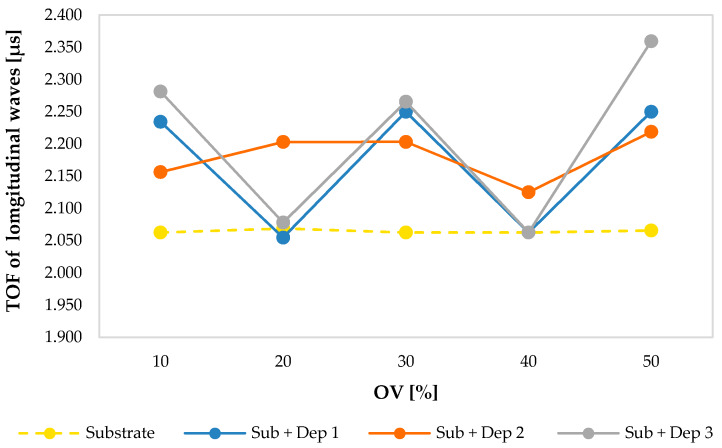
The TOF of the ultrasonic longitudinal waves propagating into the substrate and into the new material resulting by AM process (Sub + Dep_i_, for i = 1, 2, 3) measured for each specimen, i.e., as a function of the overlap percentage of the multiclad depositions.

Figure 6 shows the values of the TOF of the ultrasonic longitudinal waves that propagate in the substrate and in the new material obtained by AM process (substrate + deposition) for each sample, compared to the deposition overlap (OV) percentage. In the specimens S2 and S4, a clear deviation from the expected trend can be observed, due to the perturbation of the powder flow induced in these treatments. 

**Figure 7:**
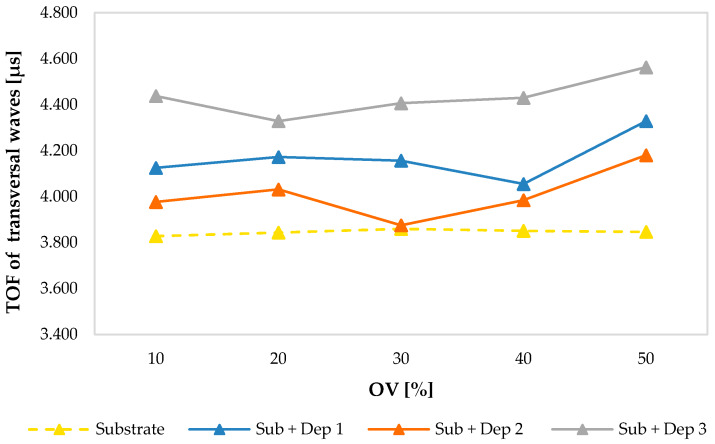
Comparison between the TOF of the ultrasonic transversal waves propagating into the substrate and into the new material resulting by AM process (Sub + Dep_i_, for i = 1, 2, 3) measured for each specimen, i.e., as a function of the overlap percentage of the multiclad depositions.

Figure 7 shows the plotted values of the TOF of the ultrasonic transversal waves that propagate in the substrate and in the new resulting AM material in relation to the deposition overlaps (OV) percentage and grouped for each set of process parameters, demonstrating a more consistent trend with a slight growth as OV increases.

In order to investigate the capability of the proposed ultrasonic approach to identify the deposition anomalies in the specimens, comparisons between the ultrasonic test results obtained in the specimens without deposition anomalies (S1, S3 and S5) and those obtained in all specimens including also those with anomalies (S2 and S4) are discussed below.

**Figure 8:**
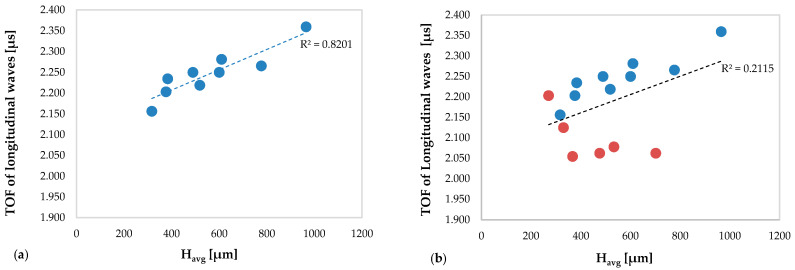
TOFs of the longitudinal waves versus H_avg_: (**a**) related to the specimens without deposition anomalies and (**b**) related to all specimens (including specimens with deposition anomalies in red color).

In particular, Figure 8 shows the TOFs of the longitudinal waves versus the average clad height obtained in the specimens without deposition anomalies (Figure 8a) and in all specimens (Figure 8b), including those with deposition anomalies. 

In Figure 8a, an excellent correlation between the values of the TOF of the longitudinal waves and the average multiclad height is observed (R^2^ = 0.8201): as the average clad height increases, the value of the TOF increases. 

By way of contrast, notice in Figure 8b the existence of a cluster of values that alter the reliability of the linear regression function. Indeed, considering also the TOF of the longitudinal waves obtained in the specimens with deposition anomalies, the close correlation between the TOF and the average multiclad height is lost (R^2^ = 0.2115). 

**Figure 9:**
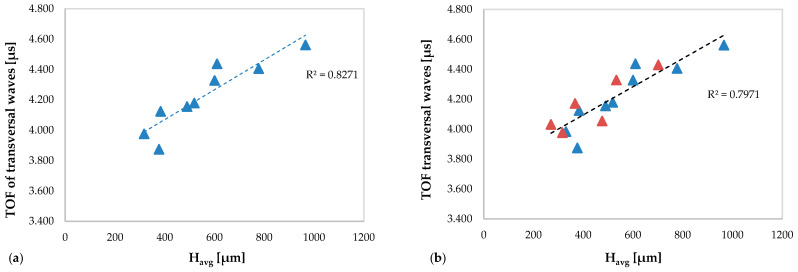
TOFs of the transversal waves versus H_avg_: (**a**) related to the specimens without deposition anomalies and (**b**) related to all specimens (including specimens with deposition anomalies in red color).

Figure 9 show the TOFs of the transversal waves versus the average height of the clad obtained in the specimens without deposition anomalies (Figure 9a) and obtained in all specimens (Figure 9b).

Figure 9a,b shows a significant correlation between the TOF of the transverse waves and the average height of the clads, but slightly more marked in the graph relating only to the specimens without defects (R^2^ = 0.8271). In both graphs, it is observed that the values of TOF of the transverse waves increases with increasing the average clad height.

It is observed that the ultrasonic results obtained in terms of the TOFs of the longitudinal waves are capable to better identify the deposition anomalies in the specimens than those obtained in terms of the TOFs of the transverse waves. 

The influence of the material mixing on the TOFs of ultrasonic waves was analyzed through Dil parameter which represents the dilution of the substrate material in the deposited clad. 

**Figure 10:**
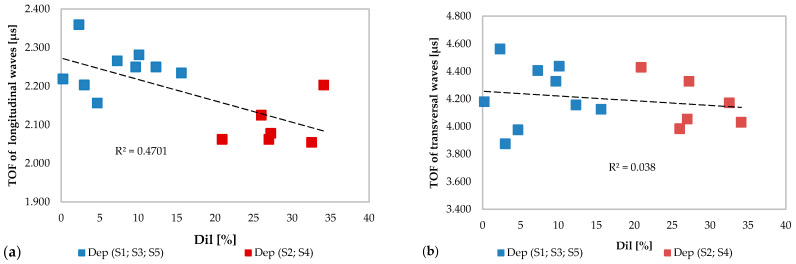
(**a**) TOFs of the ultrasonic longitudinal waves and (**b**) TOFs of the ultrasonic transversal waves versus dilution Dil grouped by deposition without anomalies (in blue color) and deposition with anomalies (in red color).

Figure 10a shows the trend of the TOF of the longitudinal waves as a function of the dilution percentage. It is noted that two separate clusters are created with a decreasing tendency to increase Dil. On the contrary, this phenomenon has no effect on the TOF of the transversal waves plotted in Figure 10b, as highlighted in the previous analysis.

By exploiting the measurement of TOFs of the ultrasonic waves in the substrate and in the new material consisting of the substrate and of the deposition, according to Figure 5, the values of ultrasonic velocities of the longitudinal waves and transversal waves, respectively, were determined. 

**Figure 11:**
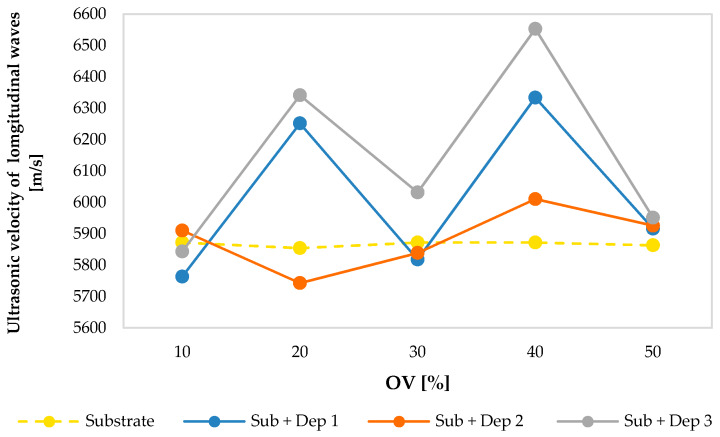
Ultrasonic velocities of the longitudinal waves of the substrate and of the new resulting material (Sub + Dep_i_, for i = 1, 2, 3) measured for each specimen, i.e., as a function of the overlap percentage of the multiclad depositions.

**Figure 12:**
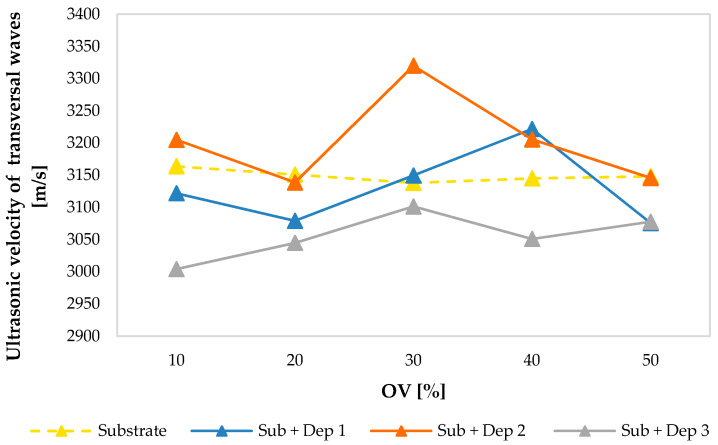
Ultrasonic velocities of the transversal waves of the substrate and of the new resulting material (Sub + Dep_i_, for i = 1, 2, 3) measured for each specimen, i.e., as a function of the overlap percentage of the multiclad depositions.

Figure 11 and Figure 12 show the values of ultrasonic velocities of the longitudinal waves (Figure 11) and of the transversal waves (Figure 12) measured in the substrate and in the new resulting material consisting of the substrate and the deposition (Sub + Dep_i_, for i = 1, 2, 3) for each specimen, as a function of the overlap (OV) percentage of the multiclad depositions.

The average values of the ultrasonic velocities of the longitudinal and of the transversal waves of the substrates are, respectively, equal to 5866.78 and 3149.22 m/s. The ultrasonic velocities measured values are comparable with those reported in the literature concerning a stainless steel AISI 304 [28,29,30]. 

As shown in Figure 11 and Figure 12, the values of the ultrasonic velocity obtained by the propagation of the ultrasonic waves in the new resulting material do not show a dependency on the overlap percentage. As already observed in Figure 6, notice that in Figure 11 the specimens S2 and S4 show a clear deviation from the expected trend of the ultrasonic longitudinal velocities. The marked deviation can be traced back to the slight perturbation of the powder flow induced in the two groups of specimens in order to evaluate the capability of the proposed ultrasonic approach for to characterization of the deposition anomalies. In Section 4, this promising capability of longitudinal waves to identify the deposition defects will be discussed.

**Figure 13:**
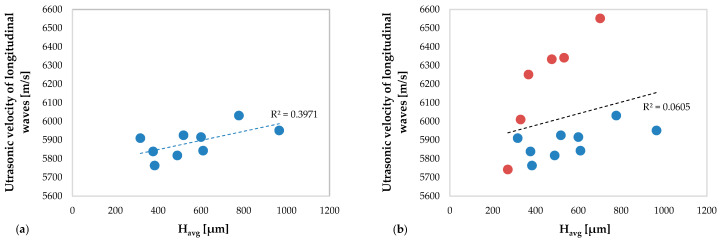
The ultrasonic velocity of the longitudinal waves versus average clad height H_avg_: (**a**) related to the specimens without deposition anomalies and (**b**) related to all specimens (including specimens with deposition anomalies in red color).

**Figure 14:**
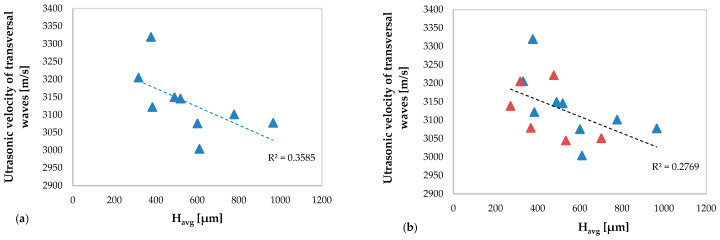
The ultrasonic velocity of the transversal waves versus average clad height H_avg_: (**a**) related to the specimens without deposition anomalies and (**b**) related to all specimens (including specimens with deposition anomalies in red color).

In Figure 13 the values of the ultrasonic velocity of the longitudinal waves obtained in the specimens without deposition defect (Figure 13a) and obtained in all specimens (Figure 13b) versus the average height of the clad are shown. The same comparison but concerning the transversal waves is shown in Figure 14.

Figure 13a and Figure 14a show a moderate correlation between the ultrasonic velocity of the longitudinal waves (R^2^ = 0.3971) and of the transversal waves (R^2^ = 0.3585), respectively, and the average clad height. In particular, the ultrasonic velocity of the longitudinal waves increases with increasing the clad height, while for the transverse waves an opposite behavior is observed. This occurs due to the different propagation characteristics (see Section 3.1) of the ultrasonic longitudinal and transversal waves, respectively, which propagate in the new material consisting of the substrate and of the deposition.

As observed in Figure 8 and Figure 9, concerning the TOFs of the ultrasonic waves, the anomalies of the depositions induced by the perturbation of the powders flow cause a variation of the ultrasonic velocity trend both of the longitudinal (R^2^ = 0.0605) and of the transverse waves (R^2^ = 0.2769). This variation is more evident in the graph relating to the ultrasonic velocity of the longitudinal waves. 

## 4. Discussion

As already highlighted in the results obtained in terms of TOF, the velocity of the longitudinal waves is capable to better identify the deposition anomalies of specimens, compared to the velocity of the transversal waves. This promising capability derives from the wave propagation features of the longitudinal waves, as the direction of motion coincides with the direction of propagation of the waves, while in transversal waves these two directions are orthogonal (see Section 3.1). 

The propagation features of the longitudinal waves allow a more accurate investigation of the variability of the mechanical behavior along the thickness of the new resulting material, which are due to the deposition process properties. Therefore, the trend of the ultrasonic velocities of the longitudinal waves increases with the increase average clad height in absence of defects, but it is strongly modified in the presence of deposition defects. 

However, the results obtained in terms of TOF of the longitudinal waves showed an even more evident correlation with the average clad height in respect to those obtained in terms of the velocity of longitudinal waves, as the times of flight are closely related to the length of the propagation path of the ultrasonic waves (i.e., the thickness of the new resulting material). 

Finally, it can be stated that the results in terms of TOF of the longitudinal waves are capable to better highlight the geometric variations of the deposition of the AM components, while the results in terms of the ultrasonic velocity of the longitudinal waves are able to show some initial information regarding the mechanical characteristics of the new materials obtained from the AM process, also highlighting the variation of the expected trend of the mechanical behavior due to process defects. 

The mechanical behavior of the AM components in terms of elastic moduli determined through the results of the ultrasonic immersion tests is analyzed below. 

Starting from the experimental values of the ultrasonic velocities, and knowing the mass density of the substrate (about 7900 kg/m^3^) and the mass density of the new materials obtained by the AM process (Table 5), applying Equation (5) has determined the engineering elastic moduli: the Young’s modulus, the shear modulus and the Poisson’s ratio for the substrate and for the new resulting material for each specimen. 

**Figure 15:**
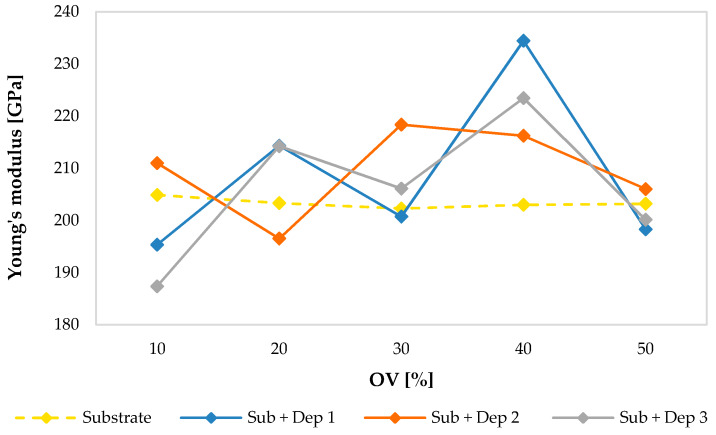
Young’s modulus values of the substrate and of the new material (Sub + Dep_i_, for i = 1, 2, 3) measured for each specimen, i.e., as a function of the overlap percentage of the multiclad depositions.

**Figure 16:**
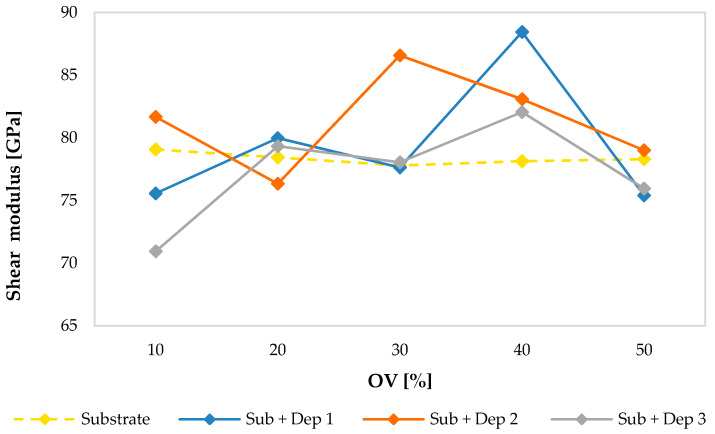
Shear modulus values of the substrate and of the new material (Sub + Dep_i_, for i = 1, 2, 3) measured for each specimen, i.e., as a function of the overlap percentage of the multiclad depositions.

**Figure 17:**
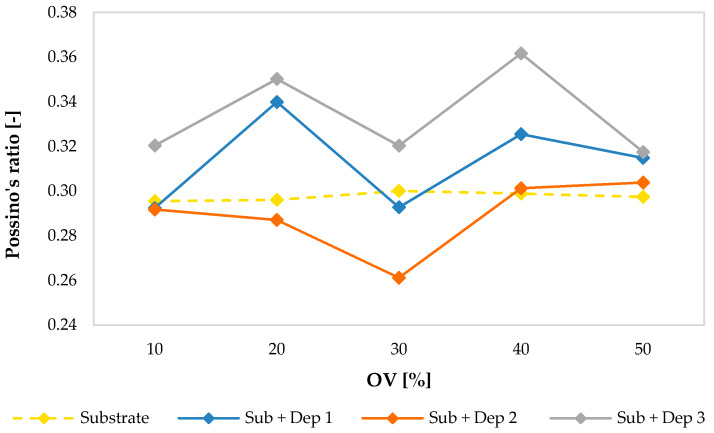
Poisson’s ratio values of the substrate and of the new resulting material (Sub + Dep_i_, for i = 1, 2, 3) measured for each specimen, i.e., as a function of the overlap percentage of the multiclad depositions.

The following graphs show the values of the Young’s modulus (Figure 15), of the shear modulus (Figure 16) and of the Poisson’s ratio (Figure 17) evaluated for the substrate and for the new material consisting of the substrate and the deposition (Sub + Dep_i_, for i = 1, 2, 3) for each specimen, as a function of the overlap (OV) percentage of the multiclad depositions.

As previously observed, regarding the values of the velocities of the ultrasonic waves, and even regarding the values of the elastic moduli of the substrate of the specimens, there were very small variations between the specimens. The average values of the Young’s modulus (203.33 GPa), of the shear modulus (78.35 GPa) and of the Poisson’s ratio (0.298) are comparable with those reported in the literature concerning a stainless steel AISI 304. 

On the contrary, the material obtained by the AM process showed a clear difference between the elastic moduli of the specimens. Moreover, as observed in the acoustic response (see Figure 8, Figure 9, Figure 11 and Figure 12), the mechanical response in terms of elastic moduli does not show a dependence on the overlap (OV) percentage of the multiclad deposition. 

Finally, the capability of the proposed ultrasonic approach to assess the deposition anomalies is investigated through the comparison of the mechanical behavior observed both in the specimens without deposition anomalies (S1, S3 and S5) and those observed in the all specimens (including the specimens with deposition anomalies) and the average height of the clads.

**Figure 18:**
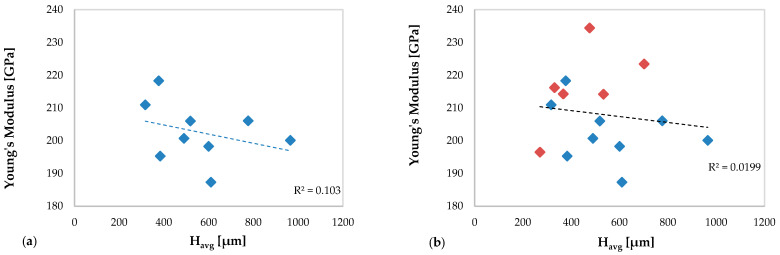
Young’s modulus values of the new resulting material versus H_avg_: (**a**) specimens without deposition anomalies and (**b**) all specimens (including specimens with deposition anomalies in red color).

Figure 18 shows the trend of the Young’s modulus as a function of the average height of the clad in specimens without deposition anomalies (Figure 18a) and in all specimens, including the specimens with deposition anomalies (Figure 18b).

In Figure 18a a low correlation (R^2^ = 0.103) between the mechanical behavior of AM specimens in terms of Young’s modulus and the average multiclad height is observed. 

In Figure 18b related to all specimens, including the specimen with anomalies of deposition, the trend of Young’s modulus considerably varies, and the correlation observed in specimens without defect completely vanishes (R^2^ = 0.0199). 

**Figure 19:**
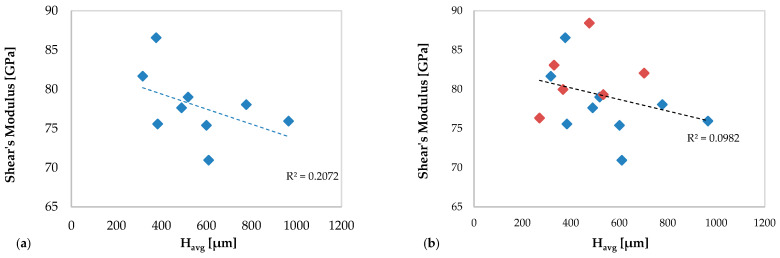
Shear’s modulus values of the new resulting material versus average clad height: (**a**) specimens without deposition anomalies and (**b**) all specimens (including specimens with deposition anomalies in red color).

In Figure 19, the correlation between the shear modulus and the average height of the clad in specimens without deposition anomalies (Figure 19a) and in all specimens (Figure 19b) are shown.

In this case, a best correlation is observed between the mechanical behavior of AM specimens in terms of the shear modulus and the average clad height in the specimens without defects (R^2^ = 0.2072), which is lost considering also the specimens with defects (R^2^ = 0.0982). 

Both graphs in Figure 18 and Figure 19 show that the values of the elastic moduli decrease as the average height of the multiclad increases.

**Figure 20:**
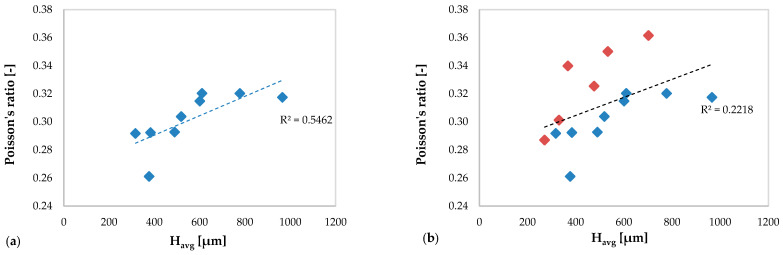
Poisson’s ratio values of the new resulting material versus average clad height: (**a**) specimens without deposition anomalies and (**b**) all specimens (including specimens with deposition anomalies in red color).

Figure 20 show the correlation between the Poisson’s ratio and the average height of the clad in specimens without deposition anomalies (Figure 20a) and in all specimens (Figure 20b).

Here, there is a closer correlation between the mechanical behavior of AM specimens in terms of the Poisson’s ratio and the average height of the multiclad compared to the previous cases, especially in Figure 20a relating to the specimens without anomalies (R^2^ = 0.5462). 

In Figure 20b a decrease of this correlation is observed (R^2^ = 0.2218) due to the values obtained in the specimens with deposition anomalies that alter the trend.

Unlike what is observed in plots relating to Young’s modulus (Figure 18) and the shear modulus (Figure 19), it is noted that the values of the Poisson ratio increase with increasing average height clad.

The results obtained in terms of elastic moduli show a lower correlation of the mechanical response of the new material resulting from the AM process from the average clad height, except those obtained in terms of Poisson’s ratio. In any case, the expected trends of moduli vary considerably, when also taking into account the results obtained in the specimens with deposition anomalies.

## 5. Conclusions

The aim of the work was to investigate the capability of the proposed ultrasonic approach on the characterization of additive manufactured components. The ultrasonic goniometric immersion tests have performed an accurate mechanical characterization of the substrate and the new material consisting of the substrate and the deposition obtained by the AM process.

According to the obtained results, it can be asserted that there is a very close correlation between the acoustic and mechanical behavior of the AM components and the average height of the clads. Exploiting this significant correlation, the effectiveness of the proposed ultrasonic approach for the characterization of deposition anomalies, expressly created in two specimens, was investigated. Indeed, the ultrasonic results related to the specimens with deposition anomalies showed an alteration of the expected trend both in the acoustic and mechanical parameters of the AM components. In particular, the results obtained in terms of the TOF and velocity of the ultrasonic longitudinal waves have better highlighted the alteration of the expected acoustic and mechanical behavior of the AM components.

On the contrary, the ultrasonic transversal waves were less affected by deposition anomalies, demonstrating a robust assessment approach and more consistent values of the average clad height in all samples, including those with defects. However, these waves did not provide any information on component deposition anomalies.

## Figures and Tables

**Table 1:** AISI304 chemical composition (ASM).

C	Cr	Ni	Mn	Si	P	S	Fe
0.08	18–20%	8.00–10.50%	2.00%	1.00%	0.045%	0.03%	66.34–74.00%

**Table 2:** Nickel-based superalloy chemical composition.

C	Cr	Mo	Co	B	Other	Ni
≤0.07%	14.6%	4.2%	15%	0.015–0.016%	7.6%	balance

**Table 3:** Process parameters.

Deposition	P	D	V	F
	[W]	[mm]	[mm/min]	[g/min]
1	600	1.00	750	5.00
2	400	1.50	750	2.50
3	600	1.00	500	5.00

**Table 4:** Specimens: denomination and geometrical characteristics of each deposition.

Specimen Overlap [%]	Deposition	W [µm]	Ac [μm^2^]	Am [μm^2^]	H_avg_ [µm]	p_avg_ [µm]	Dil [%]
**S1-OV10%**	1.1	7358	2,819,623	522,432	383	71	15.6
	1.2	5185	1,641,342	80,638	317	16	4.7
	1.3	7901	4,816,624	541,832	610	69	10.1
**S2-OV20%**	2.1	6836	2,508,826	1,210,793	367	177	32.6
	2.2	4973	1,344,553	696,406	270	140	34.1
	2.3	7463	3,979,401	1,489,496	533	200	27.2
**S3-OV30%**	3.1	6055	2,963,106	416,358	489	69	12.3
	3.2	4250	1,599,721	49,534	376	12	3.0
	3.3	6396	4,968,972	390,807	777	61	7.3
**S4-OV40%**	4.1	5449	2,593,035	959,149	476	176	27.0
	4.2	4083	1,348,843	474,142	330	116	26.0
	4.3	6043	4,240,390	1,121,606	702	186	20.9
**S5-OV50%**	5.1	4655	2,963,106	299,270	600	64	9.7
	5.2	3429	1,599,721	4227	518	1	0.2
	5.3	4978	4,968,972	113,891	965	23	2.3

**Table 5:** Mass density [kg/m^3^] of the new material (substrate + deposition) obtained by AM process.

	OV10%	OV20%	OV30%	OV40%	OV50%
**Sub + Dep 1**	7753.68	8436.28	7826.88	8420.81	7971.26
**Sub + Dep 2**	7951.35	7749.64	7855.18	8085.94	7984.08
**Sub + Dep 3**	7861.33	8457.15	8014.45	8515.42	8018.48
